# Evaluating Resonant Acoustic Mixing as a Wet Granulation Process

**DOI:** 10.1021/acs.oprd.4c00347

**Published:** 2024-12-06

**Authors:** Matthew Frederick Lopez Villena, Zachary Dean Doorenbos, Kyle Thomas Sullivan, Blair Brettmann

**Affiliations:** † Materials Science Division, Lawrence Livermore National Laboratory, Livermore, California 94550, United States; ‡ Chemical and Biomolecular Engineering, Georgia Institute of Technology, Atlanta, Georgia 30332, United States; § Materials Science and Engineering, Georgia Institute of Technology, Atlanta, Georgia 30332, United States

**Keywords:** granulation, process, agglomeration, resonant acoustic mixing

## Abstract

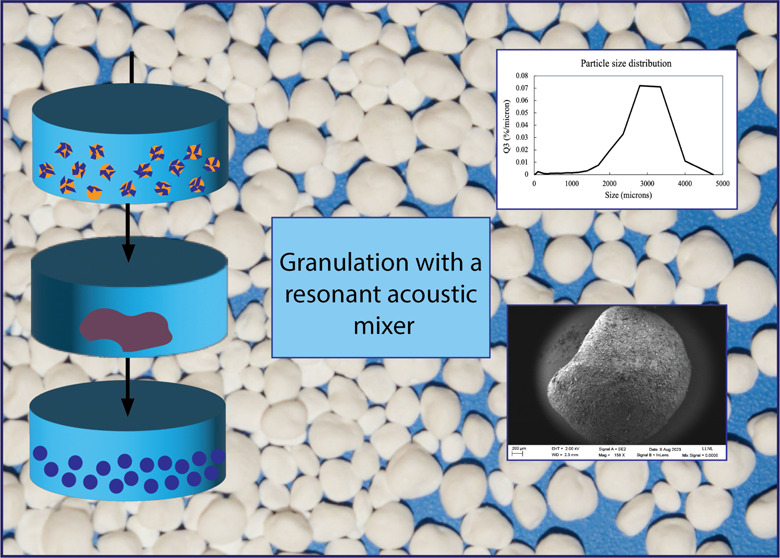

Control of powder properties is crucial for industrial processes across the food, pharmaceutical, agriculture, and mineral processing industries, and granulation is an important tool for providing agglomerated particles with controllable properties. However, existing granulation processes are not readily integrated with other processing steps and are not appropriate for some types of materials. Adding resonant acoustic-based granulation to the toolkit has the potential to widen the achievable parameter space and, importantly, integrate granulation into chemistry and blending operations that are already being performed on the RAM platform, resulting in process intensification. Here, we demonstrate the formation of granules with particle sizes of ca. 1–3 mm in LabRAM II and examine the formation mechanisms in the context of common wet granulation processes. The RAM granulation process followed here involves first forming a large “doughball” agglomerate and then driving its breakup by evaporating the solvent, while impacting the doughball against the container walls. We show that this process is similar to the destructive nucleation model for high-shear wet granulation with the solvent evaporation in our case leading to the decrease in the liquid saturation of the doughball, a corresponding decrease in its tensile strength, and the acceleration in the RAM establishing the impact pressure when the doughball contacts the walls. This work provides a foundation for granulation process design with a resonant acoustic mixer and, through its link to existing granulation mechanisms, provides a path to a deeper understanding of the process.

## 1 Introduction

Materials production with powders is important across many industries, from mineral processing to pharmaceuticals and food science to agriculture, but loose powders are challenging to work with, and often particles must be combined with a binder for the final product. Both physical and compositional properties of particles are well known to affect the handling and utility of these powders, and this is an active area of research.^1^ Thus, a number of approaches have been developed to agglomerate loose powder into granules with a controllable size distribution and material properties. Some advantages of using granules over loose powders include reduced dustiness,^2^ improved flowability,^3^ enlarged particles,^4^ controlled bulk density enabling more favorable compaction mechanics,^5^ and improved content uniformity.^6^ However, despite all of these advantages of creating granules, granulation processes add a complex unit operation to manufacturing that can be challenging to develop and control. Furthermore, some materials may be difficult to assemble or incompatible with existing granulation approaches, so alternate methods could enable new applications.

To advance the goals of process intensification, integration of the granulation with chemistry and blending steps can decrease the number of operations. One method for this is to perform granulation in a resonant acoustic mixer (RAM), which has previously been shown to be useful for powder blending,^7,8^ mixing of dense pastes,^9^ and catalytic organic synthesis.^10^ This could allow synthesis, blending, and granulation in the same unit. RAM technology agitates particles, liquids, or suspensions at the material’s resonant frequency, leading to a high degree of displacement of the mixing vessel with very efficient power consumption.^11^ The process conditions can be specifically tailored to a variety of material and manufacturing needs with parameters such as mixing intensity (acceleration relative to gravity), time, and vacuum control, making this a versatile tool. To develop a RAM granulation process, we must first understand current granulation process mechanisms and how the unique mixing environment in the RAM may fit these mechanisms. We are particularly interested in using RAM for wet granulation, where a formulation of powder and liquid binder is held together via capillary and viscous forces until the granules are dried and form strong bonds. This focus is driven by the ability of wet granulation to provide a high degree of mixing between a dissolved polymer binder and the particles. A handful of methods for wet granulation have been used in commercial industrial processes and will be discussed in greater detail in the next section. However, for many of these, it is hard to directly integrate with other steps in the manufacturing process, limiting the opportunities for process intensification.

A few studies have noted the occurrence of agglomeration during the RAM mixing processes. Windler et al. noted that prill, a type of agglomerated granule, was formed through a RAM process with three particle types, magnesium hydroxide, glycouril, and theobromine, but no details were provided on the RAM agglomeration process.^12^ In two other studies, RAM granulation processes were developed to screen for the agglomeration potential of active pharmaceutical ingredients. Interestingly, in these cases, the goal was to screen formulations that might unintentionally agglomerate during the drying process and not to develop a new granulation method. The RAM agglomeration screening method was noted to be reproducible and reduce both the time and quantity of materials required compared to the conventional method, but no attempt was made to understand and control the process.^13,14^ Finally, in a study focused on evaluating the binder styrene-ethylene/butylene-styrene (SEBS) for solid propellants, a RAM agglomeration process that occurred by mixing a wet slurry and filtering the resulting agglomerates was used.^15^ These prior studies show the potential for using RAM for agglomeration and granulation but do not consider the mechanisms or report on process development.

In this work, we demonstrate a RAM granulation process, examining the different stages in detail and putting them in context with existing wet granulation processes. To aid in adding context, we first provide a review of commercial processes, including high- and low-shear wet granulation, fluidized bed granulation, and spherical agglomeration. We discuss the common mechanisms occurring throughout the existing processes and hypothesize the mechanisms and rate processes controlling the RAM granulation process. We show that by first forming a “doughball” and then breaking it into pieces under high acceleration, we can prepare round and approximately spherical granules with average sizes ranging from 1 to 3 mm. With RAM growing as a powder mixing process, this work provides a starting approach to simplify the manufacturing process and enable both powder mixing and granulation with the same equipment.

## 2 Existing Wet Granulation and Agglomeration Processes

**Figure 1:**
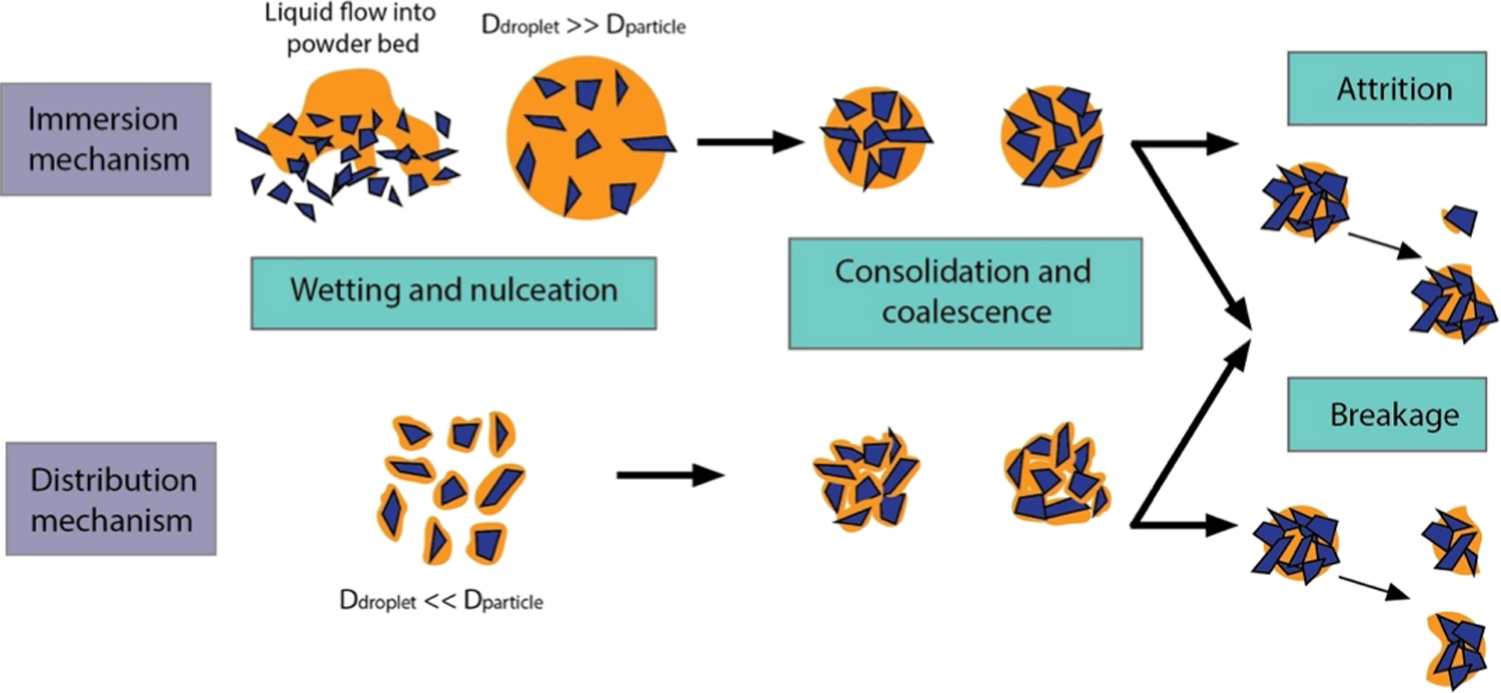
Stages of granulation including wetting and nucleation, consolidation and coalescence, and attrition and breakage. Both the immersion mechanism, where the liquid droplet size is larger than the particle, and the distribution mechanism, where the liquid droplet size is smaller than the particle size, are illustrated for wetting and nucleation and consolidation and coalescence.

Wet granulation and particle agglomeration processes share underlying mechanisms and rate processes, which are (1) wetting and nucleation, (2) coalescence and consolidation, and (3) attrition and breakage (Figure 1). These often occur simultaneously and so are not necessarily treated as three separate stages. Despite the commonalities, different granulation methods have different dominant mechanisms and rate processes and the specific material variables can also influence the relative dominance of the mechanisms. Here we briefly review four wet granulation processes for the purpose of setting the new RAM granulation process into context with the existing technologies. For an in-depth review of the mechanisms of granulation, see Iveson et al.^16^ The four processes we examine are high-shear wet granulation, low-shear wet granulation, fluidized bed granulation, and one wet agglomeration method that takes place in a continuous liquid phase, spherical agglomeration.

**Figure 2:**
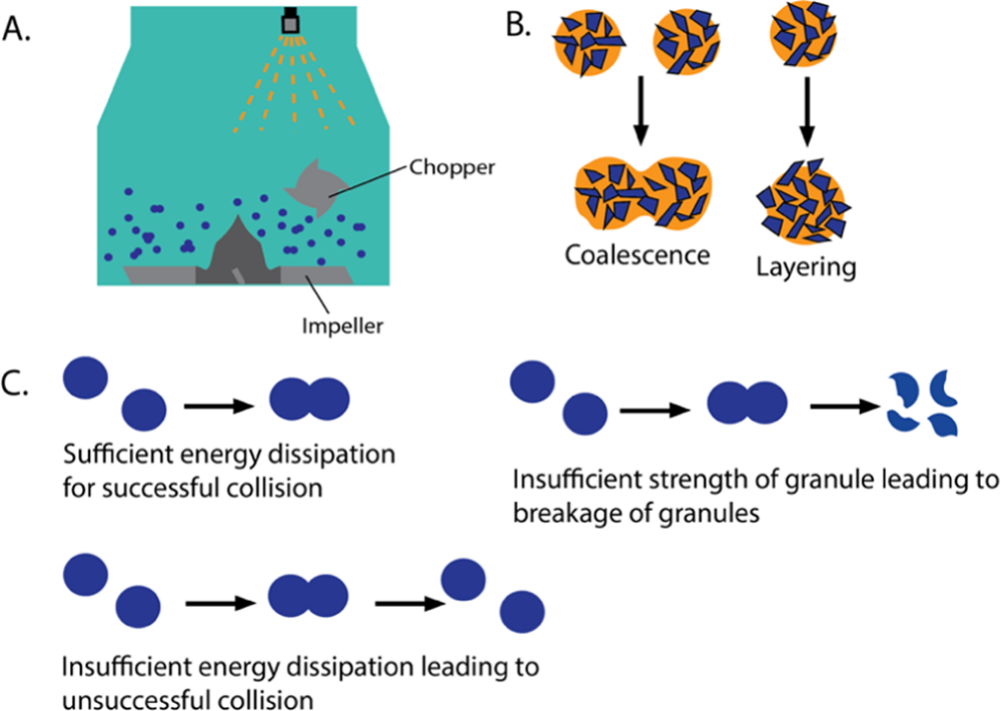
Illustration of (A) a high-shear wet granulator; (B) coalescence method of growth, where two aggregates come together, and layering, where primary particles join the surface of an existing granule; and (C) three transformations that can occur when granules collide during high-shear wet granulation.

High-shear wet granulation (HSWG) is a very common granulation process, particularly in the food^17,18^ and pharmaceutical industries.^19^ Although there are many specific designs for applying the high shear, most involve the use of a mixing bowl or cylinder and a bladed impeller that rotates at speeds from 100 to 500 rpm (Figure 2A).^19^ A chopper is also implemented to break down large agglomerates into granules. During the process, powder is loaded into the vessel and liquid binder is injected while the impeller is running at a low speed.^19^ This corresponds to the wetting and nucleation processes in Figure 1. The rest of the granulation process occurs under high-speed mixing with the impeller, with both consolidation/coalescence steps and attrition and breakage occurring during this phase.

Nucleation and growth of agglomerates in HSWG can occur through two mechanisms depending on the binder addition droplet size: when *D*_droplet_ > *D*_particle_, an immersion mechanism is followed, while for *D*_droplet_ < *D*_particle_, a distribution mechanism is followed (Figure 1).^16^ However, with HSWG, the destructive forces do not always allow the droplet/particle mixtures to come to equilibrium and follow the simplistic processes represented by these two mechanisms. Instead, it is common for breakage to occur during the wetting and nucleation stages. One model for representing the nucleation/growth process in HSWG is “destructive nucleation”,^20^ where, after initial wetting and nucleation through either the immersion or distribution mechanisms, the nucleation process continues through fragmentation of the initial agglomerates, with the fragmented pieces serving as secondary nuclei.^20^ A hallmark of HSWG is that the attrition and breakage stage of granulation is not separated from the wetting/growth or consolidation/coalescence due to the high shear forces applied, complicating the models but leading to more rapid granulation than more gentle processes.

Granule growth and consolidation often occur simultaneously and depend on the process conditions, especially the impact velocities, and on the material properties, namely, the mechanical properties of the granule and the availability of liquid binder at the surface of the granule.^16^ Growth of granules can proceed through two mechanisms: coalescence, where two large granules collide and stick together, and layering, where fines stick onto the surface of existing granules (Figure 2B). Consolidation then refers to the process of densification of the granule through reduction in porosity and removal of liquid binder.

A nucleated granule exposed to high-shear mixing can undergo a number of different transformations as the process proceeds (Figure 2C). It can collide with another granule, leading to sticking if the kinetic energy of the collision is dissipated, preventing them from rebounding. This leads to growth via coalescence and will be more dominant with large impact forces and more deformable granules, where deformation can dissipate the collision energy, and the high impact forces provide enough energy to overcome viscous and capillary forces in the granule. This dissipation will occur through viscous dissipation from the liquid layer on the surface and through plastic deformation of the granule itself.^16^ If the granules cannot dissipate the kinetic energy or the adhesion between surfaces is not strong enough to withstand future collisions, the coalescence is not permanent, and the granules will split back into two, though they often will have experienced some consolidation during the process. Another potential transformation of granules under high shear is that they collide but one or both granules break during the collision. This happens at high impact and shear forces when granule strength is low and cracks form that lead to fragmentation (Figure 2C). Larger granules are thus broken into smaller pieces, which themselves can then undergo coalescence, consolidation, or further breakage. For all of these transformations, key factors to understand and design HSWG processes include the mechanical strength of the granules, the impact forces experienced by the granules (tied to impeller design and speed), and the liquid binder properties including amount of binder, viscosity, and surface tension. The simultaneously occurring processes, which are especially complex for HSWG due to the strong breakage mechanisms make it difficult to model, but many experimental studies examine these key factors^21−23^ and provide paths for process design.

**Figure 3:**
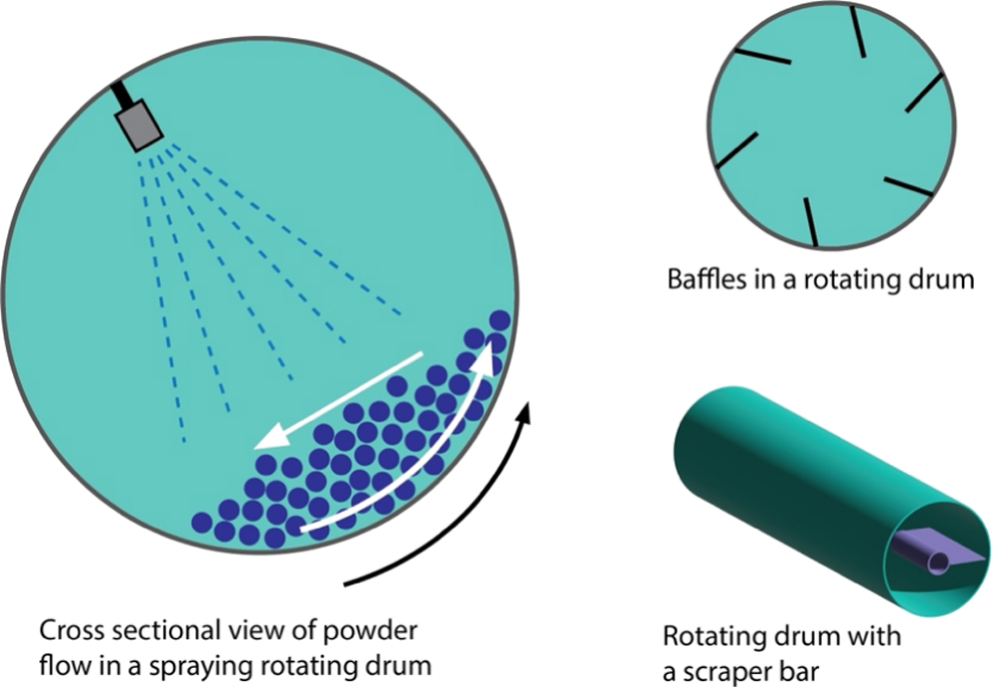
Illustration of a rotating drum granulator including a cross-sectional view showing the binder spraying and the movement of the powder, a cross-sectional view of a drum with baffles, and a 3D illustration of a rotating drum with a scraper bar.

Another approach to granulation involves applying a low shear to the mixture, which can be done through a tumbling mechanism, blenders with impellers that provide a low shear, and fluidized bed granulation. This is called low-shear wet granulation (LSWG). There is not a specific cutoff for what shear is considered low, medium, or high, but generally granulation drums, ribbon and paddle blenders, screw mixers, and basic planetary mixers have been considered as low-shear granulators.^19^ The fluidized bed granulators are also low to medium shear, though they are typically considered as a separate class and will be discussed here as such. Granulation drums are a particularly gentle mixing and agglomeration method and are frequently used in fertilizer production.^24,25^ They tumbled the material in a rotating drum, spraying the binder solution during early stages of granulation (Figure 3). Internal features such as baffles and scrapers aid the particle flow and encourage aggregation into spherical granules.^19^ Many lab-scale low-shear wet granulation studies are performed with a rotating drum granulator, so much of the published literature focuses on this type of equipment.^26−29^

As with other granulation processes, three phenomena occur during LSWG: wetting and nucleation, coalescence and consolidation, and attrition and breakage. For LSWG processes with spray addition of binder, the wetting and nucleation will depend on the droplet size, with small droplet sizes leading to the distribution mechanism, followed by coalescence (Figure 1).^30^ The consolidation and coalescence of granules during low-shear granulation processes is driven by the deformation of the granules and the sticking of the particles through the binder on the surface.^16^ With drum granulators and others with gentle particle movement, the impact velocities of the particles can be low, leading to less deformation per impact and slower granule growth. Thus, the granule mechanical properties become particularly important: with high-strength granules, the impact forces are insufficient to squeeze liquid to the surface, leading to very slow initial growth and higher growth rates once the binder does wet the surface.^31^ This is known as induction growth behavior.^32^ With low-strength granules, however, the granules will still deform under the low-impact forces and achieve steady growth.^32^ Unlike HSWG, with the lower impact forces and with fewer collisions, there is less drive for attrition and breakage, leading to stable granules with the particle size distribution mostly controlled by the consolidation and coalescence phenomena, though this will still depend on the specific formulation.^32^

**Figure 4:**
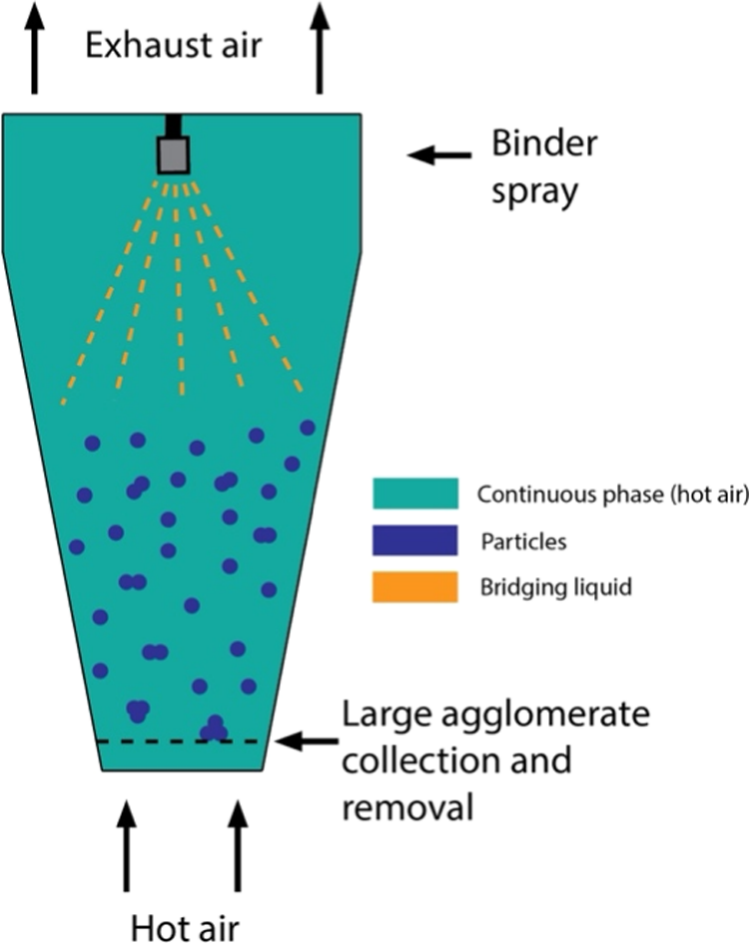
Illustration of a spray fluidized bed agglomeration process.

A specific low- to medium-shear agglomeration process is spray fluidized bed agglomeration. In this type of process, a binder liquid is sprayed onto a bed of particles that is fluidized by a gas stream, enabling agglomeration, as the particles collide and stick together. Interestingly, the spray fluidized bed technology is used in powder drying, where agglomeration is not desirable, as well as in agglomeration processes. Thus, there has been significant effort in understanding which material and processing parameters impact the onset of agglomeration and development of models to predict this point.^33,34^ In many fluidized bed agglomeration processes, the particles are loaded into the bed, and hot air flows from the bottom to top, fluidizing the particles. A liquid binder is sprayed from the top, interacting with the particles in the bed volume. As particles agglomerate, they gain mass and thus are more likely to settle to the bottom of the bed, where they can be removed, providing a method to separate agglomerates once they reach a critical size. Figure 4 illustrates the process.

In spray fluidized bed granulation, particles aggregate due to adhesion to a liquid layer. The binder droplets must first contact and spread on the particles and then the particles must collide and the adhesion force during the collision must be strong enough to prevent the particles from rebounding and separating.^33^ Put in the context of the typical mechanisms in Figure 1, the binder wetting the particles follows a distribution mechanism; then, coalescence occurs when the particles collide and stick. As the impact forces are not high in this process, there is little consolidation of the granules, typically leading to less dense granules than other methods.^35^ There also is typically only a low amount of attrition and breakage, especially since drying of the binder occurs throughout the process due to the flowing hot air, leaving polymer binder strongly bridging the particles. Since most of the granule properties, such as size distribution, sphericity, and density, are driven by the wetting and coalescence of droplets, the most important process and material parameters to consider are related to the binder wetting and spreading on the particle and the probability of two particles impacting and sticking together. This has been modeled through consideration of the probability of wet collisions (that two particles that are wet collide with one another) and that those collisions are “successful” (they result in the particles staying together).^33^ Through experiments and models, it has been shown that the key parameters influencing agglomerate formation and size are the inlet gas temperature and air velocity, the droplet size used, the viscosity of the binder, and its contact angle on the particles.^33,36^

**Figure 5:**
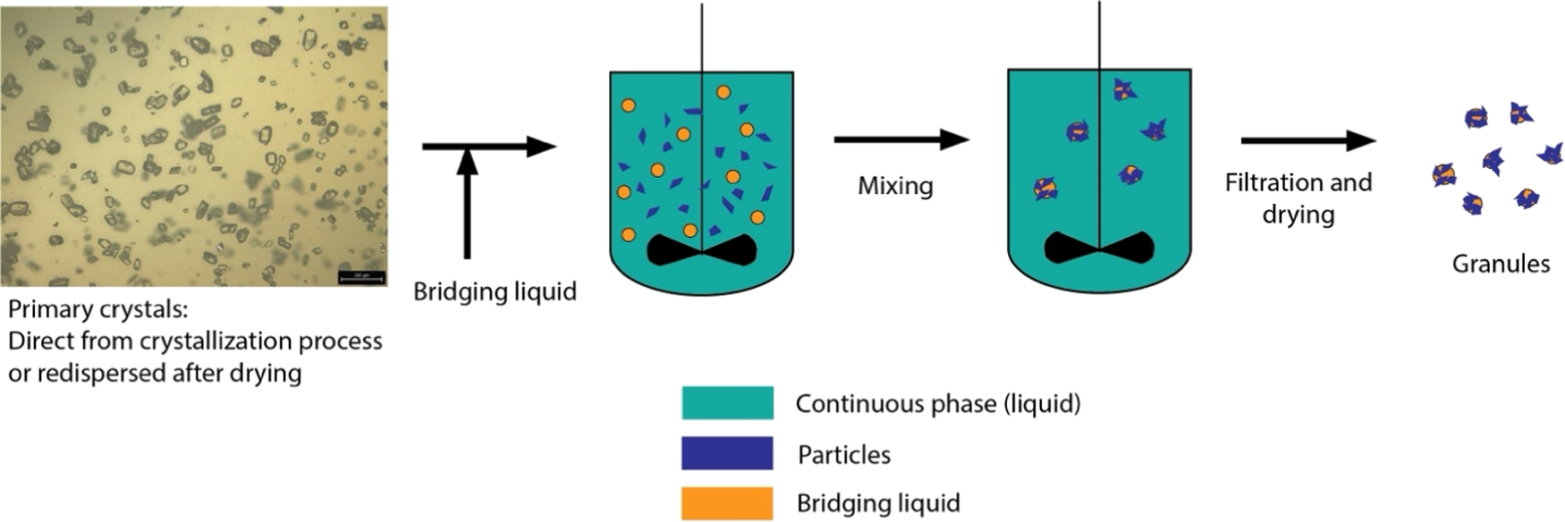
Illustration of spherical agglomeration process from primary crystals to granules.

In contrast to the methods discussed so far, spherical agglomeration, also called “agglomeration in suspension” and “spherical crystallization” occurs with particles dispersed in a continuous liquid phase. Where spray fluidized bed agglomeration relies on droplets impacting crystals in air and cohesion between those particles, spherical agglomeration uses immiscible liquid droplets in the continuous phase that preferentially interact with the particle surfaces, leading to coating and particle cohesion. There are two advantages to agglomeration in suspension: (1) it can be used immediately after crystallization without needing to dry the particles, a time-consuming and energy-intensive process, and (2) it reduces the breakage and attrition mechanism of granulation processes, leading to fewer residual fines. Figure 5 illustrates a typical spherical agglomeration process that occurs after the crystallization process. Similar to other granulation processes, the system undergoes nucleation/wetting, growth and coalescence, consolidation and breakage/attrition and population balance models have been used to predict the resulting agglomerate size distribution.^37,38^

The most important design consideration for spherical agglomeration is the bridging liquid, the immiscible fluid that is used to agglomerate the particles. It must have a good wettability of the particle, but also be immiscible in the continuous phase.^39^ In addition to the wetting and miscibility criteria, another important design parameter is the amount of binding liquid. If too little is used, agglomeration will not occur, but if too much is used the agglomerates are soft and paste-like.^39^ Thus, for each process, there is an optimum ratio of binding liquid to the particles. As an example, for a system with lobenzarit disodium particles, *n*-hexane as the binding liquid and water as the continuous phase, the minimum binder-to-solids ratio for spherical agglomerates is 1.08 and the maximum ratio is 1.43.^39^ Although most spherical agglomeration processes are performed with solely solvents and particles, there have been some examples where a polymer is also incorporated into the agglomerates. This is typically done by dissolving the polymer in the continuous liquid and can lead to different morphologies, such as more rounded granules or large irregular agglomerates, depending on the polymer selected,^40,41^ though little work has been done to fully understand the mechanism of the dissolved polymer joining the growing agglomerates.

As noted in the above discussion, the mechanisms shown in Figure 1 for wet agglomeration and granulation are common for existing processes, though they exhibit different dominant mechanisms and rates. This variety provides the ability to granulate powders with different materials, obtain granules with different properties and to integrate with other manufacturing technologies. Adding resonant acoustic-based granulation to the toolkit has the potential to widen the achievable parameter space, and, importantly, integrate granulation into chemistry and blending operations that are already being performed on the RAM platform,^7,9,10^ resulting in process intensification. However, to achieve this, we must first identify a procedure with RAM that can produce granules and identify the governing mechanisms for the process, enabling comparison with existing processes and providing routes for process design.

## 3 Results

**Figure 6:**
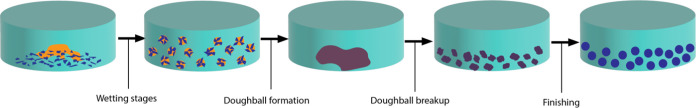
Illustration of the stages of the RAM granulation process.

A resonant acoustic mixer (LabRAM II) equipped with a mixing vessel with a diameter-to-height ratio of 0.4 and a vacuum attachment was used to prepare granules (Figure S1A shows a photograph of the setup). The granulation occurred through a doughball breakup method, where the particles and fluids are mixed such that they form one large agglomerate, a “doughball,” and then the doughball is broken up into smaller agglomerates (illustrated in Figure 6, doughball photo in Figure S1B). This is in contrast to the prior study that used a RAM to prepare agglomerates (molding powder),^15^ which used a solvent addition to suspension approach, similar to the spherical agglomeration method. Our doughball approach was used to decrease solvent usage while still enabling good mixing between the binder solution and the particles. In the RAM granulation process used here, there are four processing stages that are run one after another (without user intervention), and the transitions are illustrated in Figure 6. The first is a wetting stage, which occurs at low accelerations and without applying a vacuum. The second is a stage for the formation of a doughball, which happens at moderate accelerations and low vacuum pressure. In the third stage, the doughball is broken up through further moderate acceleration and low vacuum pressures, leading to well-mixed agglomerates. In this stage, some RAM processing time is included postbreakup at the same conditions to ensure the doughball breaks into sufficiently small pieces and that those pieces lose most of their solvent. In the final stage, high acceleration is used with high vacuum pressure to obtain desired particle properties such as density and sphericity and to finish the drying process.

**Figure 7:**
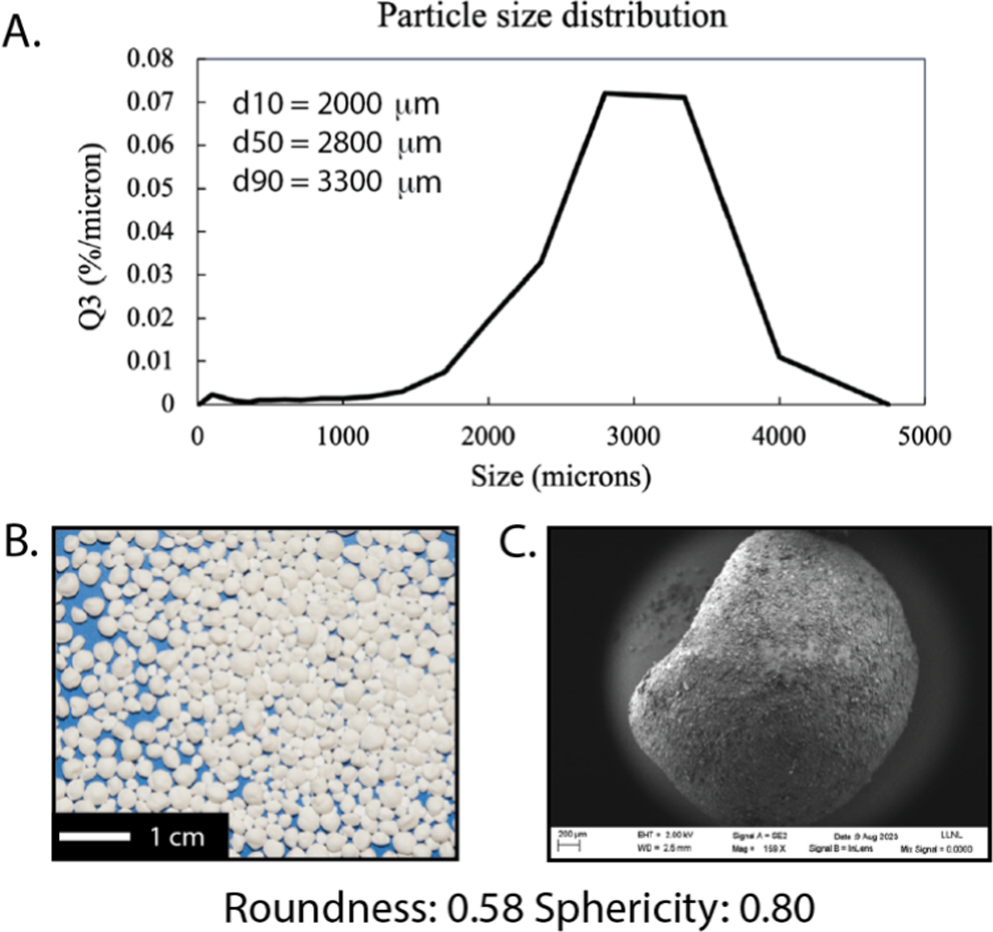
(A) Particle size distribution as measured with a Camsizer, (B) photograph and (C) SEM image of granules produced with the conditions in Table 1.

To demonstrate this process, we prepared a mixture that was 69 wt % melamine fines (primary particle size distribution and SEM image in Figure S2) in a polymer binder solution that was 10 wt % fluoropolymer elastomer in ethyl acetate. Using the RAM process conditions in Table 1, we obtained a yield of 41.9% granules with a sieve mesh size of 1080 μm with no measurable amount of fines. The remaining material (51.1% of the starting material) was adhered to the base and top of the container used for mixing (photo in Figure S3). The granules had a particle size distribution with a *d*_10_ of 2000 μm, a *d*_50_ of 2800 μm, and a *d*_90_ of 3300 μm (Figure 7A) and had relatively isotropic shapes that were irregular and not smoothly spherical with a roundness mean value of 0.58 and a sphericity of 0.80 as measured through the CamSizer (SEM image and photo in Figure 7B,C). Although these are large for some applications, such as pharmaceuticals, they are in range for others such as fertilizers^42^ and the tuning of particle size is not a main focus of this work, though would be important for further study.

**Table 1:** Baseline RAM Process for Melamine/Fluoropolymer Elastomer Granules

stage	time	acceleration	vacuum pressure
wetting 1	30 s	10*g*	N/A
wetting 2	50 s	20*g*	N/A
wetting 3	40 s	30*g*	N/A
doughball formation	3 min	60*g*	50 Torr (6666 Pa)
doughball breakup	4 min	60*g*	20 Torr (2666 Pa)
finishing	2:30 min	80*g*	600 Torr (79,993 Pa)

Thus, far, we have demonstrated that we can achieve granulation in a LabRAM II, but to better put it in context with existing granulation processes, we also analyze a key part of the process. We will focus primarily on the breakup of the doughball, which is unique to our methodology. As there are no impellers in the RAM, the breakup is driven by the impact of the doughball with the walls of the container due to acceleration. The wide and short jar used for granulation enables frequent impacts with the container, aiding in breakup and utilizing both the upper and lower transducers in the LabRAM II. Countering these impact forces is the strength of the doughball, which is expected to be affected by many parameters, including the liquid saturation level, particle size, porosity, etc. During the doughball breakup stage of the RAM granulation process, we are actively changing the liquid saturation level by applying a vacuum, driving down the doughball strength until it fractures on impact. To demonstrate this, we performed the RAM granulation process with different vacuum pressure levels during the doughball breakup step. We noted the time of the initial breakup of the large doughball and then continued the doughball breakup step conditions for another 3:25 min to ensure breakup into sufficiently small pieces before moving to the finishing step. This ensures that the only time parameter that changed was the time from the start of the doughball breakup step to the initial breakup. A summary of the overall process is shown in Table 2.

**Table 2:** RAM Process to Study the Effect of Vacuum Pressure Level on the Doughball Breakup

stage	time	acceleration	vacuum pressure level
wetting 1	30 s	10*g*	N/A
wetting 2	50 s	20*g*	N/A
wetting 3	40 s	30*g*	N/A
doughball formation	3 min	60*g*	50 Torr (6666 Pa)
doughball breakup	until initial breakup	60*g*	20–50 Torr (2666–6666 Pa)
doughball breakup 2	3:25 min	60*g*	20–50 Torr (2666–6666 Pa)
finishing	2:30 min	80*g*	600 Torr (79,993 Pa)

**Figure 8:**
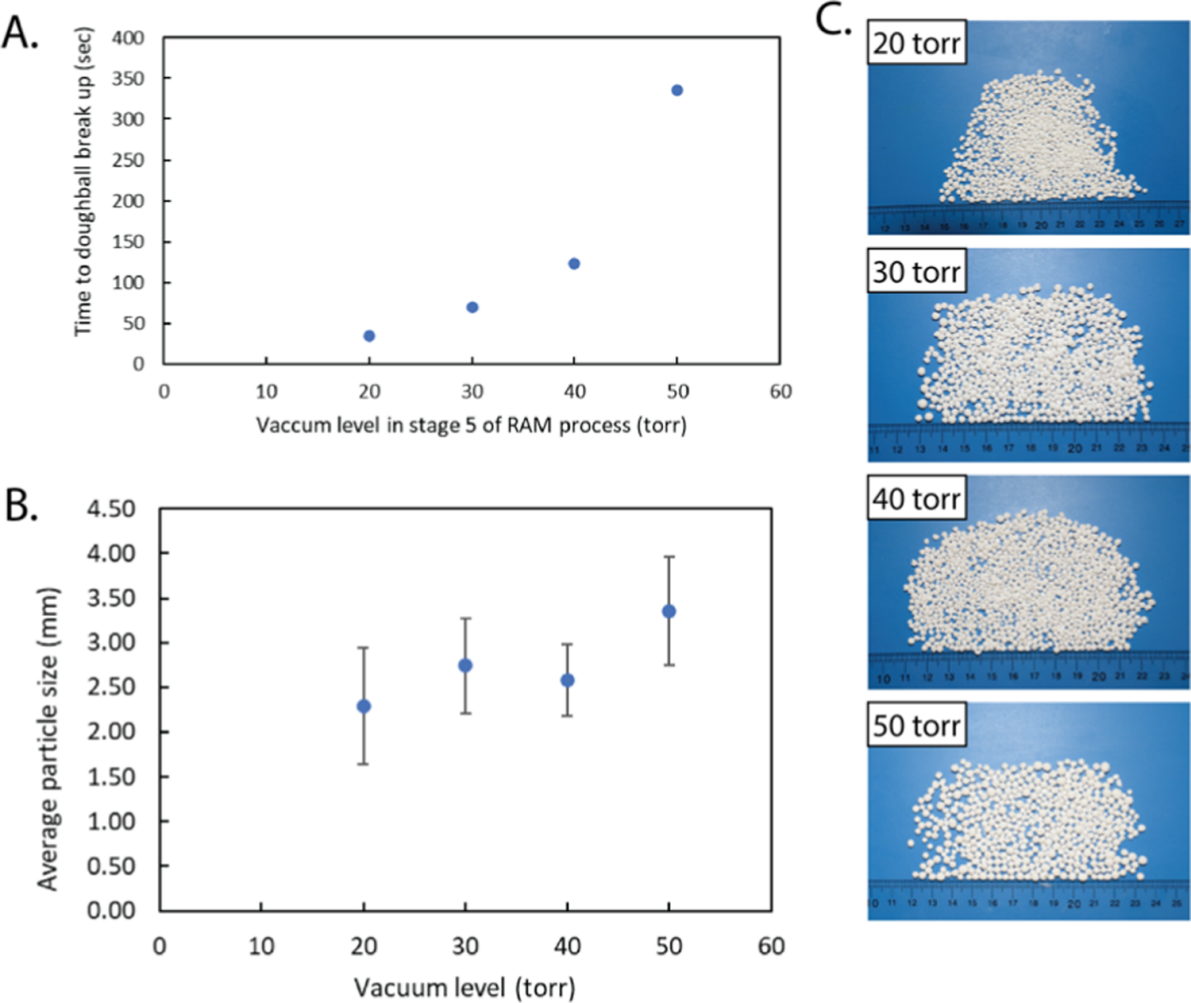
(A) Time from the start of the doughball breakup stage to the point of the breakup as a function of the vacuum pressure during that stage. (B) Average particle size as a function of the vacuum pressure during the doughball breakup stage. Measurements made through manual image analysis of images in (C) and error bars are the standard deviation of 50 measurements. (C) Photographs of granules formed with different vacuum pressures at the doughball breakup stage.

Figure 8A shows the time to initial breakup (time zero is the start of the doughball breakup step) as a function of the vacuum level during this step. As the vacuum pressure increases, the evaporation rate will be lower and the liquid level higher for a given time point. A higher liquid level leads to a stronger doughball due to the capillary forces between the particles, and thus, the doughball will resist breakup at higher liquid levels. This leads to longer times before breakup for slower evaporation processes (higher vacuum pressure). Interestingly, the change in doughball breakup time with a vacuum pressure is roughly exponential. This will be examined in more detail in the Section 4.

In addition to monitoring the time to breakup, we analyzed the particle size resulting from the four different vacuum pressures (Figure 8B) and examined images (Figure 8C) and SEM images (Figure S4) of the granules. The granules are all relatively spherical and round with a rough surface texture. Although the trend is not strong, we do see that higher vacuum pressures (50 Torr (6666 Pa)) lead to larger granules than lower vacuum pressures (20 Torr (2666 Pa)). This could be due to greater consolidation during the extra time that the doughball is impacting the walls and thus a breakup into larger pieces at those slower evaporating levels. However, given the complexity of many competing drivers for granule size, further study would be needed to determine the mechanism for this difference in granule size.

When considering the breakup of the doughball, the removal of liquid through a vacuum is an important driver for breakup, as shown above. However, the impact pressure that the doughball experiences as it collides with the bottom and top of the jar is also expected to influence the time to doughball breakup. To test this, we compared processes with 60 and 70*g* acceleration during the doughball breakup step. We found that the time to breakup at 60*g* acceleration was 2:03 min, while the time to breakup for 70*g* acceleration was 0:42 min, indicating that the higher acceleration, which leads to higher impact forces with the top and bottom of the jar, leads to faster breakup. The resulting granules had a similar average particle size and morphology, as seen in Figure S5.

Although it is outside the scope of this work to fully investigate the finishing process, we will address one aspect as it relates to the yield. It is important to maximize the yield of granules, here defined as agglomerates with sizes greater than 1 mm (greater than 1080 μm mesh), but within the RAM granulation process there are two primary methods by which the yield decreases: (1) coating of the jar and lid with solidified particle/binder mixture (seen in Figure S3) and (2) breakage and attrition of formed granules. For the first point, studies are ongoing to fully determine the link between processing and material parameters and the jar coating phenomenon, but preliminary work indicates that lowering the liquid level can decrease the lost yield from the jar coating. The finishing process specifically impacts the second of these, and we demonstrated that a higher vacuum pressure during a finishing process drastically decreases the fines produced during RAM granulation. We compared a process with a longer process time at the conditions for doughball breakup (low vacuum pressure, moderate acceleration) to one with some time spent at a higher vacuum pressure and higher acceleration (process parameters in Table 3).

**Table 3:** RAM Process to Study the Effect of Vacuum Pressure during the Finishing Step on the Granule Yield

	low vacuum pressure finishing conditions			high vacuum pressure finishing conditions		
stage	time	acceleration	vacuum level	time	acceleration	vacuum level
wetting 1	30 s	10*g*	N/A	30 s	10*g*	N/A
wetting 2	50 s	20*g*	N/A	50 s	20*g*	N/A
wetting 3	40 s	30*g*	N/A	40 s	30*g*	N/A
doughball formation	3 min	60*g*	50 Torr (6666 Pa)	3 min	60*g*	50 Torr (6666 Pa)
doughball breakup	4 min	60*g*	20 Torr (2666 Pa)	4 min	60*g*	20 Torr (2666 Pa)
finishing	5 min	60*g*	20 Torr (2666 Pa)	2:30 min	80*g*	600 Torr (79,993 Pa)

**Figure 9:**
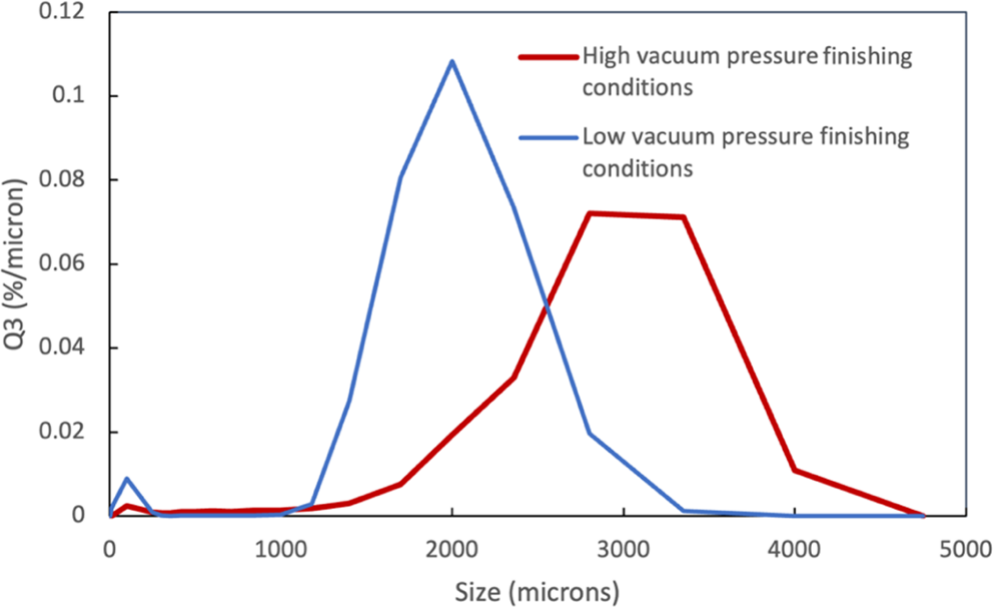
Particle size distribution as measured using a CamSizer for runs with a low vacuum pressure finishing condition (20 Torr (2666 Pa)) and high vacuum pressure finishing conditions (600 Torr (73,993 Pa)).

The yield of the process subjected to low vacuum pressure finishing conditions was 26.2% granules >1 mm, but, importantly, there were 31.8% fines. In contrast, the process with high vacuum pressure finishing conditions had a yield of 41.9% granules >1 mm and no fines produced. Figure 9 shows the particle size distribution data for the granules captured on the 1080 μm sieve formed from the low vacuum pressure finishing conditions (blue) and the high vacuum pressure finishing conditions (red). As expected, the low vacuum pressure conditions led to lower particle sizes, likely due to the breakage and attrition that resulted in the large proportion of fines.

## 4 Discussion

The process described here for granulation using acoustic mixing involves a number of processing stages, all within the same container and without user intervention. An initial gentle process (low acceleration, no vacuum) wets the particles with the binder liquid through the immersion mechanism discussed in prior granulation literature.^16^ The liquid in these stages is never broken up into small droplets, so it does not reach the distribution mechanism. Although this study focused on a single system of melamine particles with a fluoropolymer elastomer binder in ethyl acetate solvent, we anticipate that the specific parameters needed for the wetting stage will depend on the interactions between the solvent and the particle as well as the binder viscosity, similar to the design of other wetting processes.

Mixing of the liquid binder and particles continued in the doughball formation stage. This is a stage that does not have a clear equivalent in any existing mixing processes, though it can be considered as the formation of one very large granule, which is avoided in standard processes but is essentially an extreme imbalance in the rates of coalescence (high) and attrition/breakage (low). The vacuum is used during this stage to drive off some solvent and move the mixture to a regime where a doughball forms, as the liquid binder amount is kept higher for the first stages to facilitate wetting. If the liquid is not driven off, the mixture may instead form a paste, which is not readily broken into agglomerates. However, a balance must be struck, as when too strong of a vacuum is used, the material can get stuck to the bottom and top of the container. We found the doughball formation to be very consistent for this formulation and so did not investigate it in detail, but we anticipate that the vacuum pressure, acceleration, and time needed will vary with the formulation and initial amount of the solvent.

The success of this RAM granulation process depends on the breakup of the doughball into smaller agglomerates. To drive this process, we use vacuum to lower the liquid saturation level and a high acceleration to drive the impact of the doughball with the container. Ideally, this is a breakage process with minimal attrition to prevent the formation of fines. It is similar to the destructive nucleation process described by Vonk et al., where a primary nucleus, in our case the large doughball, breaks into smaller pieces that persist as granules or can form new nuclei.^20^ In that work, the breakup is described as a balance of the primary agglomerate tensile strength and impact pressure:
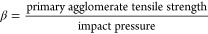
1

In Vonk et al., the breakup of the primary agglomerate occurs when β ≈ 1. The primary agglomerate tensile strength can be estimated as
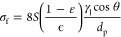
2where *S* is the liquid saturation level, ε is the porosity of the granule, γ_l_ is the binder surface tension, θ is the contact angle between the liquid and solid, and *d*_p_ is the diameter of the primary particles. The impact pressure can be estimated through:
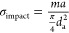
3where *m* is the mass of the agglomerate, *a* is the acceleration, and *d*_a_ is the diameter of the primary agglomerate. We can examine our results from the doughball breakup tests in the context of this model. When the vacuum is turned on, the liquid saturation, *S*, will decrease significantly, leading to a lower primary agglomerate tensile strength. The impact pressure will not change as significantly with decreasing liquid content because the agglomerate mass and diameter will change only slightly. This leads to a decrease in β as the liquid saturation level is lowered, and eventual breakup.

By testing vacuum pressures from 20 to 50 Torr (2666–6666 Pa), we also change the rate at which the liquid is driven out, and we can see that the decrease in time to breakup with a lower vacuum pressure (Figure 8) is consistent with driving forces for breakup shown in this model. However, we observed an exponential relationship, not a linear relationship, with *S* as shown in eq 2. This is likely due to the other parameters that change with time in the process such as the porosity, which will decrease with decreasing liquid saturation and impacts with the walls, and the evaporation rate. Although thermodynamically the liquid evaporation may be expected to follow a linear trend (i.e., if Raoult’s law holds), the migration of liquid to the solid/air interface and the complex surface morphology may complicate that rate. Future work could be undertaken to more specifically represent the change in liquid saturation with time and integrate this into a more precise model to describe the process.

In addition to the agglomerate tensile strength, the impact pressure also plays a role in the breakup of the doughball. Here again, our results are consistent with the model. We showed that an increase in the acceleration led to a decrease in the time to breakup, which aligns with the model’s prediction of higher accelerations leading to higher impact pressure, again driving down the value of β and leading to breakup. This shows that the destructive nucleation model is a good starting point for understanding and predicting doughball breakup in the RAM granulation process.

## 5 Conclusions

We have demonstrated the granulation of melamine with a fluoropolymer elastomer binder using a process based on resonant acoustic mixing. The process shows several similar mechanisms to other wet granulation processes but is reliant on the formation of a large doughball that subsequently breaks up as the liquid saturation is decreased. We suggest that the doughball formation and breakup mechanism is similar to the destructive nucleation mechanism described previously but at the extremely large size of a nucleated agglomerate. We show that this breakup takes longer when we pull less vacuum during the breakup stage, which is consistent with a higher agglomerate strength with higher liquid saturation levels. Additionally, the breakup takes longer at lower accelerations, where there is a lower impact pressure on the walls. Overall, the granulation process in the LabRAM II is promising for the formation of millimeter-sized granules, and we can analyze the process using existing mechanisms from other wet granulation processes. Although this is a batch process, Resodyn has developed continuous acoustic mixing systems that are promising for continuous operation and would further enable integration of this process with others in process intensification. For batch operation, the scaling of this process beyond the LabRAM II has not yet been studied, but Resodyn mixers are commercially available that can operate with 5 kg (OmniRAM), 36 kg (RAM 5), and 420 kg (RAM 55) so there is opportunity for production at larger scales. This work provides a foundation for developing integrated chemistry-blending-granulation processes in LabRAM and is promising for process intensification in powder engineering.

## 6 Materials and Methods

### 6.1 Materials

Melamine was purchased from Alfa Aesar and its particle size distribution is shown in Figure S1. Ethyl acetate was purchased from VWR and a fluoropolymer elastomer was purchased from Arkema. The binder solution was prepared by dissolving the fluoropolymer in ethyl acetate at a concentration of 10 wt % polymer.

### 6.2 RAM Granulation Process

Granules were prepared by using a multistep process in a Resodyn LabRAM II with a custom-built fixture to house a wider jar than the standard fixture. The jar used for mixing was 3D printed from VeroClear resin on a Stratasys printer and had a diameter/height ratio of 0.4. A custom-printed lid with a vacuum attachment was placed on the jar prior to granulation and attached to the LabRAM vacuum system. Due to the solvent removal during the granulation process, a solvent trap was installed between LabRAM II and the vacuum system to trap the ethyl acetate.

Powder was placed in the jar, and binder solution was added immediately prior to placing in the LabRAM II to minimize undesired solvent evaporation. Typical batch sizes used 15 g of powder. The jar with the vacuum attachment lid was installed in the assembly on the LabRAM II plate, and the process started. Although many parameters were examined in the course of this study, they generally consisted of 3 mixing steps, 1 doughball formation step, 1 doughball breakup step, and 1 finishing step, each with different run times, accelerations, and vacuum levels. When the process was finished, the jar was removed and the resulting material sieved with a 1080 μm mesh to obtain the yield of granules that meet the required particle size. If small fines were present, then the amount of small fines was also noted. The remaining solid material was adhered to the surface of the mixing jar.

### 6.3 Particle Size Analysis

For particle size analysis of granules, a CamSizer Camsizer X2 was used where noted with a 1.5–3 min run using the X-fall module. For manual image analysis, photographs of the granules were imported into ImageJ and 50 particle diameters were measured. A ruler in the photographs was used to convert pixels to millimeters. The melamine particle size distribution was measured using a Saturn DigiSizer II. Approximately 40 mg of Melamine powder was dispersed in 2 mL of Sedisperse media with 10 drops of A11 surfactant and sonicated for 3 min prior to measurement. A flow rate of 9 L/min was used with a circulation time of 120 s.

### 6.4 Scanning Electron Microscopy

Samples were gold-coated three times at 20 mA for 30 s each. SEM is the Zeiss Gemini, 30 μm aperture, *z* height 48 mm, EHT at 2.00 kV.

## Supporting Information Available

Photographs of the setup in the LabRAM, doughballs, and material adhered to the jar, particle size analysis and SEM image of particles, SEM images of granules, and additional photographs and SEM images of granules (PDF)

## References

[ref1] Fitzpatrick J. Powder Properties in Food Production Systems. In Handbook of Food Powders, 2nd ed.; Bhandari B.; Bansal N.; Zhang M.; Schuck P., Eds.; Woodhead Publishing Series in Food Science, Technology and Nutrition; Woodhead Publishing, 2024; Chapter 13, pp 203–218.

[ref2] Jensen K. A.; Koponen I. K.; Clausen P. A.; Schneider T. Dustiness Behaviour of Loose and Compacted Bentonite and Organoclay Powders: What Is the Difference in Exposure Risk?. J. Nanopart. Res. 2009, 11 (1), 133–146. 10.1007/s11051-008-9420-1.

[ref3] Schiano S.; Chen L.; Wu C.-Y. The Effect of Dry Granulation on Flow Behaviour of Pharmaceutical Powders during Die Filling. Powder Technol. 2018, 337, 78–83. 10.1016/j.powtec.2017.08.064.

[ref4] Bardin M.; Knight P. C.; Seville J. P. K. On Control of Particle Size Distribution in Granulation Using High-Shear Mixers. Powder Technol. 2004, 140 (3), 169–175. 10.1016/j.powtec.2004.03.003.

[ref5] Van Den Ban S.; Goodwin D. J. The Impact of Granule Density on Tabletting and Pharmaceutical Product Performance. Pharm. Res. 2017, 34 (5), 1002–1011. 10.1007/s11095-017-2115-5. 28188541

[ref6] Oka S.; Smrčka D.; Kataria A.; Emady H.; Muzzio F.; Štěpánek F.; Ramachandran R. Analysis of the Origins of Content Non-Uniformity in High-Shear Wet Granulation. Int. J. Pharm. 2017, 528 (1–2), 578–585. 10.1016/j.ijpharm.2017.06.034. 28627457

[ref7] Gupta S.; Pu Y. E.; Li M.; Li Z.; Osorio J. G. Assessment of Resonant Acoustic Mixing for Low-Dose Pharmaceutical Powder Blends. AAPS PharmSciTech 2022, 23 (5), 126 10.1208/s12249-022-02262-4. 35474151

[ref8] Beckel E.; Oyler K.; Mehta N.; Khatri N.; Marin J.; Shah A.; Cordaro-Gioia E.; Decker R.; Grau H.; Stec D. Primary Explosive Processing in the Resonant Acoustic Mixer. Propellants, Explos., Pyrotech. 2021, 46 (5), 697–704. 10.1002/prep.202100008.

[ref9] Kline D. J.; Grapes M. D.; Avalos E. A.; Landeros C. M.; Martinez H. P.; Reeves R. V.; Sullivan K. T.; Doorenbos Z. D. Probing the Role of Solids Loading and Mix Procedure on the Properties of Acoustically Mixed Materials for Additive Manufacturing. Powder Technol. 2022, 411, 117947 10.1016/j.powtec.2022.117947.

[ref10] Gonnet L.; Lennox C. B.; Do J.; Malvestiti I.; Koenig S. G.; Nagapudi K.; Friščić T. Metal-Catalyzed Organic Reactions by Resonant Acoustic Mixing. Angew. Chem. 2022, 134 (13), e202115030 10.1002/ange.202115030. 35138018

[ref11] How RAM Mixes. Resodyn Acoustic Mixers. https://resodynmixers.com/how-ram-mixes/ (accessed December 18, 2023).

[ref12] Windler G.; Hartline E.; Armenta C.; Berchtold K.; Bolme C.; Golder A.; Matteson J.; Moore D.; Pacheco A.; Pierce T.; Ramos K.; Saavedra R. New PBX 9501 Dynamic Mock Candidates via the Wet Slurry Process; LA-UR-20-28598, 2020.

[ref13] Zhang S.; Lamberto D. J. Development of New Laboratory Tools for Assessment of Granulation Behavior During Bulk Active Pharmaceutical Ingredient Drying. J. Pharm. Sci. 2014, 103 (1), 152–160. 10.1002/jps.23762. 24338750

[ref14] Papageorgiou C. D.; Langston M.; Hicks F.; am Ende D.; Martin E.; Rothstein S.; Salan J.; Muir R. Development of Screening Methodology for the Assessment of the Agglomeration Potential of APIs. Org. Process Res. Dev. 2016, 20 (8), 1500–1508. 10.1021/acs.oprd.6b00201.

[ref15] Wilkinson P. J.; Weaver M. C.; Kister G.; Gill P. P. Styrene-Ethylene/Butylene-Styrene (SEBS) Block Copolymer Binder for Solid Propellants. Propellants, Explos., Pyrotech. 2022, 47 (1), e202100142 10.1002/prep.202100142.

[ref16] Iveson S. M.; Litster J. D.; Hapgood K.; Ennis B. J. Nucleation, Growth and Breakage Phenomena in Agitated Wet Granulation Processes: A Review. Powder Technol. 2001, 117 (1–2), 3–39. 10.1016/S0032-5910(01)00313-8.

[ref17] Pathare P. B.; Byrne E. P. Application of Wet Granulation Processes for Granola Breakfast Cereal Production. Food Eng. Rev. 2011, 3 (3), 189–201. 10.1007/s12393-011-9043-7.

[ref18] Santomaso A. C.; Baggio R.; Zorzi F.; Salviulo G.; Realdon N.; Franceschinis E. Sugars with Different Thickening Power in High Shear Granulation. Powder Technol. 2017, 317, 391–399. 10.1016/j.powtec.2017.05.017.

[ref19] Parikh D. Handbook of Pharmaceutical Granulation Technology, 3rd ed.; Informa Healthcare: USA, 2010.

[ref20] Vonk P.; Guillaume C. P. F.; Ramaker J. F.; Vromans H.; Kossen N. W. F. Growth Mechanisms of High-Shear Pelletisation. Int. J. Pharm. 1997, 157, 93–102. 10.1016/S0378-5173(97)00232-9.

[ref21] Shi L.; Feng Y.; Sun C. C. Roles of Granule Size in Over-Granulation During High Shear Wet Granulation. J. Pharm. Sci. 2010, 99 (8), 3322–3325. 10.1002/jps.22118. 20232456

[ref22] Chitu T. M.; Oulahna D.; Hemati M. Wet Granulation in Laboratory Scale High Shear Mixers: Effect of Binder Properties. Powder Technol. 2011, 206 (1), 25–33. 10.1016/j.powtec.2010.07.012.

[ref23] Osei-Yeboah F.; Feng Y.; Sun C. C. Evolution of Structure and Properties of Granules Containing Microcrystalline Cellulose and Polyvinylpyrrolidone During High-Shear Wet Granulation. J. Pharm. Sci. 2014, 103 (1), 207–215. 10.1002/jps.23776. 24218097

[ref24] Adetayo A. A.; Litster J. D.; Desai M. The Effect of Process Parameters on Drum Granulation of Fertilizers with Broad Size Distributors. Chem. Eng. Sci. 1993, 48 (23), 3951–3961. 10.1016/0009-2509(93)80374-Y.

[ref25] Walker G. M.; Holland C. R.; Ahmad M. N.; Fox J. N.; Kells A. G. Drum Granulation of NPK Fertilizers. Powder Technol. 2000, 107 (3), 282–288. 10.1016/S0032-5910(99)00253-3.

[ref26] De Simone V.; Caccavo D.; Lamberti G.; d’Amore M.; Barba A. A. Wet-Granulation Process: Phenomenological Analysis and Process Parameters Optimization. Powder Technol. 2018, 340, 411–419. 10.1016/j.powtec.2018.09.053.

[ref27] De Simone V. D.; Dalmoro A.; Lamberti G.; d’Amore M.; Angela Barba A. Central Composite Design in HPMC Granulation and Correlations between Product Properties and Process Parameters. New J. Chem. 2017, 41 (14), 6504–6513. 10.1039/C7NJ01280B.

[ref28] Mills P. J. T.; Seville J. P. K.; Knight P. C.; Adams M. J. The Effect of Binder Viscosity on Particle Agglomeration in a Low Shear Mixer/Agglomerator. Powder Technol. 2000, 113 (1), 140–147. 10.1016/S0032-5910(00)00224-2.

[ref29] Iveson S. M.; Litster J. D. Fundamental Studies of Granule Consolidation Part 2: Quantifying the Effects of Particle and Binder Properties. Powder Technol. 1998, 99 (3), 243–250. 10.1016/S0032-5910(98)00116-8.

[ref30] Schæfer T.; Mathiesen C. Melt Pelletization in a High Shear Mixer. IX. Effects of Binder Particle Size. Int. J. Pharm. 1996, 139 (1), 139–148. 10.1016/0378-5173(96)04548-6.

[ref31] Wauters P. A. L.; Van De Water R.; Litster J. D.; Meesters G. M. H.; Scarlett B. Growth and Compaction Behaviour of Copper Concentrate Granules in a Rotating Drum. Powder Technol. 2002, 124 (3), 230–237. 10.1016/S0032-5910(02)00029-3.

[ref32] Iveson S. M.; Litster J. D. Growth Regime Map for Liquid-Bound Granules. AIChE J. 1998, 44 (7), 1510–1518. 10.1002/aic.690440705.

[ref33] Rieck C.; Bück A.; Tsotsas E. Estimation of the Dominant Size Enlargement Mechanism in Spray Fluidized Bed Processes. AIChE J. 2020, 66 (5), e16920 10.1002/aic.16920.

[ref34] Khadilkar A.; Rozelle P. L.; Pisupati S. V. Models of Agglomerate Growth in Fluidized Bed Reactors: Critical Review, Status and Applications. Powder Technol. 2014, 264, 216–228. 10.1016/j.powtec.2014.04.063.

[ref35] Gao J. Z. H.; Jain A.; Motheram R.; Gray D. B.; Hussain M. A. Fluid Bed Granulation of a Poorly Water Soluble, Low Density, Micronized Drug: Comparison with High Shear Granulation. Int. J. Pharm. 2002, 237 (1–2), 1–14. 10.1016/S0378-5173(01)00982-6. 11955799

[ref36] Strenzke G.; Dürr R.; Bück A.; Tsotsas E. Influence of Operating Parameters on Process Behavior and Product Quality in Continuous Spray Fluidized Bed Agglomeration. Powder Technol. 2020, 375, 210–220. 10.1016/j.powtec.2020.07.083.

[ref37] Peña R.; Burcham C. L.; Jarmer D. J.; Ramkrishna D.; Nagy Z. K. Modeling and Optimization of Spherical Agglomeration in Suspension through a Coupled Population Balance Model. Chem. Eng. Sci. 2017, 167, 66–77. 10.1016/j.ces.2017.03.055.

[ref38] Blandin A. F.; Mangin D.; Subero-Couroyer C.; Rivoire A.; Klein J. P.; Bossoutrot J. M. Modelling of Agglomeration in Suspension: Application to Salicylic Acid Microparticles. Powder Technol. 2005, 156 (1), 19–33. 10.1016/j.powtec.2005.05.049.

[ref39] Amaro-González D.; Biscans B. Spherical Agglomeration during Crystallization of an Active Pharmaceutical Ingredient. Powder Technol. 2002, 128 (2), 188–194. 10.1016/S0032-5910(02)00196-1.

[ref40] Jitkar S.; Thipparaboina R.; Chavan R. B.; Shastri N. R. Spherical Agglomeration of Platy Crystals: Curious Case of Etodolac. Cryst. Growth Des. 2016, 16 (7), 4034–4042. 10.1021/acs.cgd.6b00563.

[ref41] Thakur A.; Thipparaboina R.; Kumar D.; Gouthami K. S.; Shastri N. R. Crystal Engineered Albendazole with Improved Dissolution and Material Attributes. CrystEngComm 2016, 18 (9), 1489–1494. 10.1039/C5CE02306H.

[ref42] Pamungkas R. B.; Jos B.; Djaeni M.; Saputri K. A. D. Granulation Processing Variables on the Physical Properties of Granule Slow Release Urea Fertilizer. AIP Conf. Proc. 2020, 2197 (1), 100001 10.1063/1.5140952.

