# Supplementary material for: Evaluating Resonant Acoustic Mixing as a Wet Granulation Process
Source: Org Process Res Dev. 2024 Dec 6;28(12):4338–47. doi: 10.1021/acs.oprd.4c00347 (PMC11667745; doi:10.1021/acs.oprd.4c00347)
Supplement: Supplementary file 1 — op4c00347_si_001.pdf [file op4c00347_si_001.pdf]

Supporting Information

Evaluating resonant acoustic mixing as a wet granulation process

Matthew Frederick Lopez Villena[1], Zachary Dean Doorenbos[1], Kyle Thomas Sullivan[1], Blair Brettmann[1,2,3]

1) Materials Science Division, Lawrence Livermore National Laboratory, Livermore, CA, 94550
2) Chemical and Biomolecular Engineering, Georgia Institute of Technology, Atlanta, GA, 30332
3) Materials Science and Engineering, Georgia Institute of Technology, Atlanta, GA, 30332

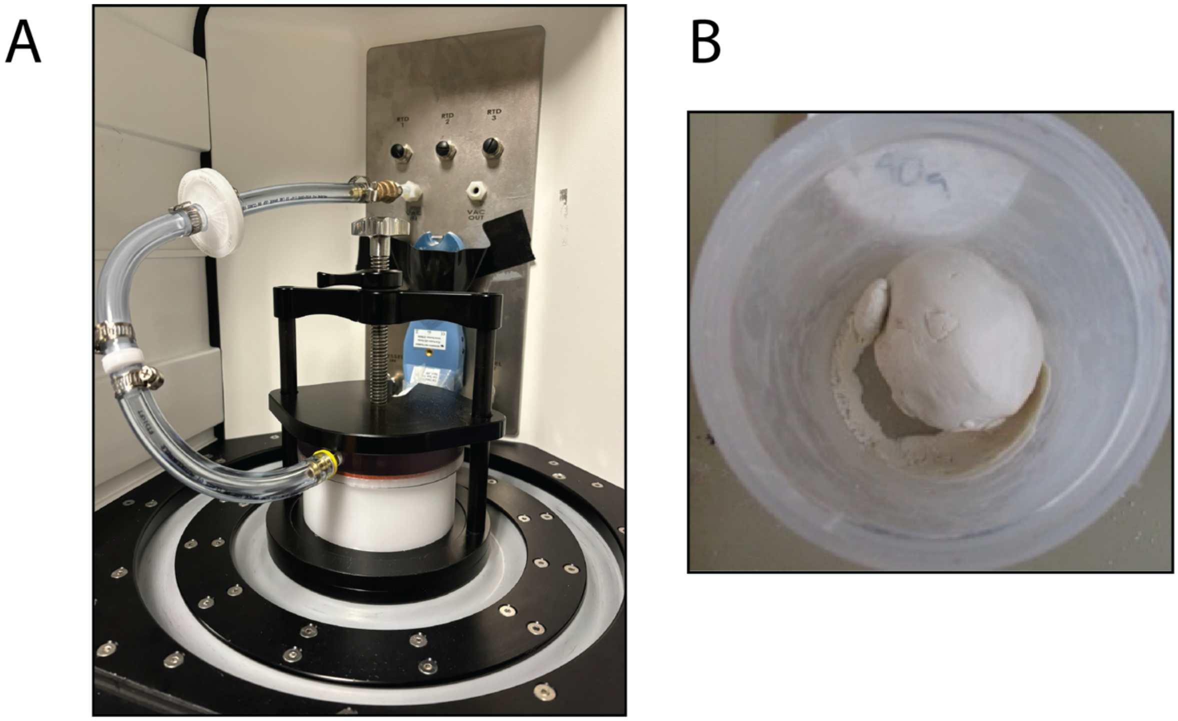

Figure S1: A) A representative LabRAM set up for the powder agglomeration process. The vessel containing powder is positioned between the plates and tubing to the vacuum line extends from a lid on the vessel to an exit port on the LabRAM. B) Photograph of a doughball in a jar.

A. 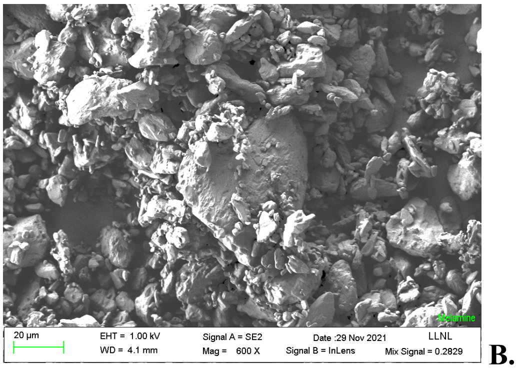

B. 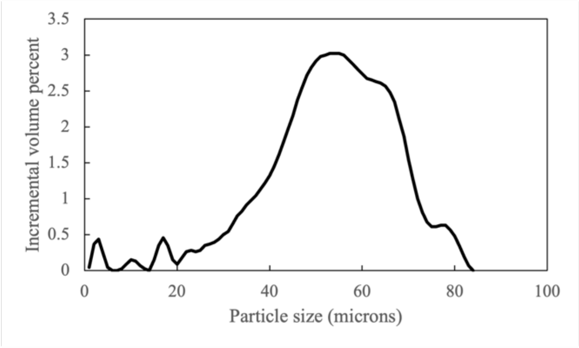

Figure S2: A) Scanning electron microscopy image of melamine particles, B) Particle size distribution of Melamine primary particles as measured through laser scattering.

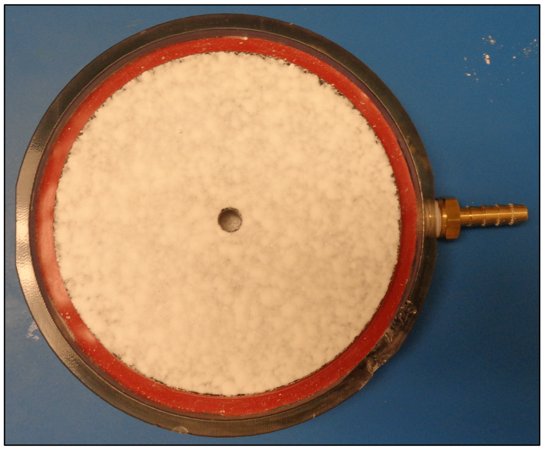

Figure S3: Melamine and binder adhered to lid of jar after granulation with the LabRAM

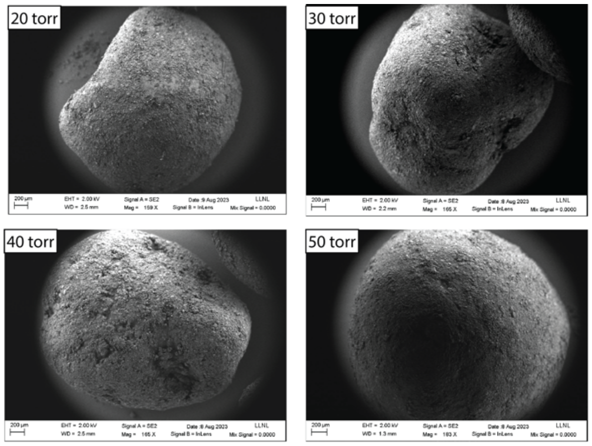

Figure S4: SEM images of granules formed through doughball breakup process with different vacuum pressures during the doughball breakup stage.

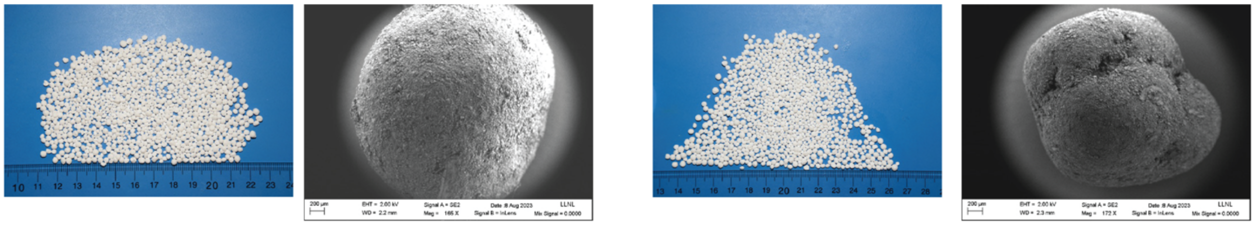

Avg dia (mm)	2.58
stdev	0.40

Avg dia (mm)	2.39
stdev	0.58

Figure S5: Photographs and SEM images of granules prepared using the doughball breakup method with 60 g and 70 g accelerations during the doughball breakup step. Also shown are the average particle diameters and standard deviations as measured through manual image analysis with 50 measurements.
